# The Impact of Cancer Status on Anxiety in Prostate Cancer Patients: A Network Analysis

**DOI:** 10.3390/curroncol31120566

**Published:** 2024-12-01

**Authors:** Christopher F. Sharpley, Kirstan A. Vessey, Vicki Bitsika, Wayne M. Arnold, David R. H. Christie

**Affiliations:** 1 Brain-Behaviour Research Group, University of New England, Armidale, NSW 2351, Australia; kvessey@une.edu.au (K.A.V.); vicki.bitsika@une.edu.au (V.B.); warnold3@une.edu.au (W.M.A.); david.christie@genesiscare.com (D.R.H.C.); 2 GenesisCare, John Flynn Hospital, Tugun, QLD 4224, Australia

**Keywords:** prostate cancer, anxiety, network

## Abstract

Prostate cancer (PCa) patients often also suffer from comorbid anxiety, which can impede treatment efficacy as well as be intrinsically unpleasant. Identification of the associations between particular symptoms of anxiety that are most likely to occur at different points in the PCa diagnosis–treatment journey can inform anxiety treatment choices and potentially influence their overall treatment outcomes. Although simple correlational analyses and ANOVA models of data analysis have been used to address this issue, the possibility of confounds due to the inter-relationships between other anxiety symptoms argues for the use of network analysis, which calculates each symptom–symptom connection while also taking into account the entire range of symptom relationships. Responses to the GAD-10 self-report scale for Generalised Anxiety Disorder were collected from 415 PCa patients who were grouped according to whether (1) their PCa was just diagnosed and undergoing initial treatment; (2) their cancer was in remission; or (3) their cancer was recurring after initial treatment. The results of the network analysis indicated several areas where clinically relevant differences were present between the three PCa groups, but caution was applied to the results of statistical tests due to unequal sample sizes. Individual GAD symptom–symptom association differences are discussed in terms of their implications for directed and individualised anxiety-management treatment models.

## 1. Introduction

Prostate cancer (PCa) patients often suffer from anxiety [1], which is observed in as many as 28% of all PCa patients [2]. As well as being intrinsically unpleasant, this anxiety can reduce the overall quality of patients’ lives and impair PCa treatment outcomes [1]. Consequently, there is clinical benefit in being able to understand the nature of anxiety in these men, plus how factors such as the patients’ cancer status and treatments are associated with patient anxiety.

The most common method of determining associations between anxiety and clinical factors is correlational analysis. However, network analysis allows for those correlations to be depicted graphically and enables each association between (for example) anxiety and cancer status/treatment experiences to be depicted after taking into account all other associations in the variable matrix, which is not possible under the usual correlational models of data analysis [3]. Network analysis also allows for a description of the causal interactions between symptoms, as well as the ways in which those symptoms may be associated via feedback loops [4].

This is particularly important when considering the most common form of anxiety (Generalised Anxiety Disorder—GAD) because that disorder is diagnosed by the presence of eight quite heterogeneous clinical criteria, including excessive, uncontrollable worry about a variety of topics most days; distress and/or functional impairment; and somatic symptoms such as restlessness or feeling keyed up or on edge, being easily fatigued, difficulty concentrating or having one’s mind go blank, irritability, muscle tension, and sleep disturbance [5]. Previous studies of the networks between depression and alleviating factors in PCa patients have been reported [6], but no previous report has been made of the ways in which PCa patients’ cancer status is related to their GAD.

Because it has been previously established that the impact of cancer status is linked with PCa patients’ anxiety [7], it is relevant to the delivery of appropriate care to these men to investigate the nature of the associations between their cancer status/treatment regimes and their GAD. In particular, a comparison between PCa patients currently undergoing initial treatments versus those whose treatment had finished and who were in remission versus those whose PCa had been treated but had recurred was undertaken in this study because it might be expected that these different stages of PCa could be associated with different levels of GAD severity and networks. Because GAD is heterogeneous, and those different GAD symptoms require different psychological treatments, this investigation also needs to be undertaken at the level of individual GAD symptoms as well as the GAD total score level.

Therefore, this study examined the networks between GAD symptoms for each of the three different points in the PCa diagnosis and treatment process (referred to here as ‘cancer status’). The aim of the study was to firstly describe those networks and then compare them across three stages of PCa diagnosis and treatment. It was hoped that these results would enable some implications for the development of effective anti-anxiety treatments for those PCa patients to be drawn. Because there is no previous report of comparison between the GAD networks of PCa patients at varying stages of their treatment, a null hypothesis approach was assumed here (i.e., there would be no difference between the network structure of PCa patients when identified according to their cancer status).

## 2. Materials and Methods

### 2.1. Participants

The PCa patients in this study were volunteers from three treatment centres in south-east Queensland. Inclusion criteria were as follows: patients had had their PCa established via biopsy, were currently undergoing treatment, and either had current cancer or were in remission after treatment; all patients also needed to have discussed their treatment options with their GP, a radiation oncologist, or a urologist and be fluent in English. Section 2.3 describes that these patients were verbally invited to participate in a study ‘about how you feel’ by their treating oncologist. They were then offered the questionnaire by the clinical reception staff and asked to return the completed questionnaire.

### 2.2. Measures

PCa patients were asked to complete an English language background questionnaire (age, PCa status, date of first diagnosis) and the GAD-10 for how they felt at the time of completing the questionnaire. Because there was a range of current and past treatments (surgery, radiotherapy, hormone therapy, plus all combinations of these, and no treatment), providing very small cell sizes for some treatments, treatment type was not able to be examined in any valid way.

The GAD-10 is an extended version of the GAD-7, which includes some of the diagnostic criteria for GAD from the Diagnostic and Statistical Manual of Mental Disorders (5th ed, Text Revision) (DSM-5-TR) [5]. Although the GAD-7 has excellent internal consistency (Cronbach alpha = 0.92) and good test–retest reliability (intraclass correlation = 0.83), it included only seven items. To ensure that all of the GAD diagnostic criteria were included, three more items were added to the GAD-7 in this study (covering symptoms of sleep difficulties, concentration difficulties, and fatigue) to form the GAD-10.

### 2.3. Procedure

A total of 558 PCa patients received an open verbal invitation from their treating oncologist to participate in a research project ‘about how you feel’. Those who accepted this invitation (unknown by their oncologist) were given a package of questionnaires described above by reception staff including a Participant Information Statement and a Consent Form for signing, all of which were to be returned to the reception staff at the clinic they attended. The participation rate for usable data was 74.3% (*n =* 415). This study was approved by the Uniting Care Health Human Research Ethics Committee (approval number and date 11 December 2023) in accordance with the Helsinki Declaration of 1964 and confirmed in 2013. Written informed consent was obtained from all participants.

### 2.4. Statistical Analyses

SPSS 29 was used to derive descriptive statistics, plus the internal consistency (Cronbach’s alpha) for the GAD-10. The normality of the GAD-10 was determined via the Kolmogorov–Smirnov statistic and inspection of the detrended normal Q–Q plots and histograms. Spearman correlation coefficients and ANOVA were used to detect significant associations between age, time since diagnosis, cancer status, and the GAD-10, with appropriate Bonferroni corrections to the *p* value to reduce the likelihood of Type I error due to family-wise error rate.

Network analysis was used to determine correlations between GAD-10 items in prostate cancer patients with cancer present, cancer recurring after initial treatment, or in remission. This analysis was completed using R studio run through JASP 0.19.1.0 freeware (https://jasp-stats.org/ (accessed on 25 October 2024)). These network structures were determined using an initial Spearman correlation matrix, followed by analysis using the Extended Bayesian Information Criterion with a graphical lasso algorithm (EBICglasso) from the *bootnet* package. ‘Nodes’ were defined for the ten GAD-10 items for the prostate cancer patients undergoing their first treatment for PCa, those whose PCa was recurring after initial treatment, and those men whose PCa was in remission. ‘Edges’ were the associations between each pair of these nodes.

The 10 GAD-10 items (with abbreviations) were as follows: 1, Feeling nervous, anxious, on edge (Nerv); 2, Unable to control worrying (Contr); 3, Worrying too much about different things (Worry); 4, Trouble relaxing (Relax); 5, Restless (Restl); 6, Easily annoyed, irritable (Irrit); 7, Afraid something awful will happen (Afraid); 8, Easily Tired (Tired); 9, Difficulty concentrating (Conc); and 10, Difficulty sleeping (Sleep). Edges between nodes are based on partial correlations between any two nodes, after controlling for all other nodes in the network. Edge weights are the strength of the connections. Edge weights were plotted using a heatmap to show the unique influence of one node on another, while controlling for all other nodes using a custom script in Python 3.13.0. A comparison of the three networks was undertaken by several procedures including structural comparisons, visualization, and the Network Comparison Test [8].

## 3. Results

### 3.1. Data

The PCa patients were aged between 48 and 84 years (*M* = 67.9 yr, SD = 6.5 yr). Patients were screened so that they had all received their PCa diagnosis no more than six years previous to the study (*M* = 31.3 mo, SD = 21.2 mo), to contribute to the homogeneity of the sample. Their GAD-10 scores ranged from 10 to 32 (*M* = 13.9, SD = 4.4) out of a possible maximum score of 40. Because GAD-10 total scores were found to be non-normally distributed, Spearman correlation coefficients were used to determine that there were no significant correlations between participants’ ages and their GAD-10 total scores (*ρ* = −0.014, *p* = 0.806) or between their time since diagnosis and their GAD-10 total score (*ρ* = −0.041, *p* = 0.466).

Patients’ current cancer status was as follows: cancer present and receiving initial treatment (*n =* 71, 17.2%), cancer in remission (n = 300, 72.1%), or cancer recurring after treatment (*n =* 44, 10.7%). PCa patients whose cancer was recurring after treatment had significantly higher GAD-10 total scores (*M* = 16.57, SD = 5.31) than men whose PCa was in remission (*M* = 13.44, SD = 4.06), *F*(1,253) = 15.655, *p* < 0.001, partial eta squared = 0.058. Although those PCa patients whose cancer was currently being initially treated had GAD-10 scores (*M* = 14.74, SD = 4.65) that were higher than those of PCa patients whose cancer was in remission (*M* = 13.44, SD = 4.06), *F*(1,273) = 4.079, *p* = 0.044, that difference was not significant at the corrected *p* level of 0.05/3 = 0.016. Similarly, patients whose cancer was recurring after treatment had higher GAD-10 total scores than patients whose PCa was currently undergoing initial treatment (*F*(1,96) = 4.806, *p* = 0.031), but that difference did not meet the corrected level of significance. However, the major aim of this study was to investigate the effect of cancer status upon the association between GAD-10 items for each cancer-status subgroup rather than the total GAD-10 score and how these individual GAD-1 item associations may have differed between cancer stage groups.

### 3.2. Network Structures

#### 3.2.1. PCa Present and Receiving Initial Treatment

**Figure 1:**
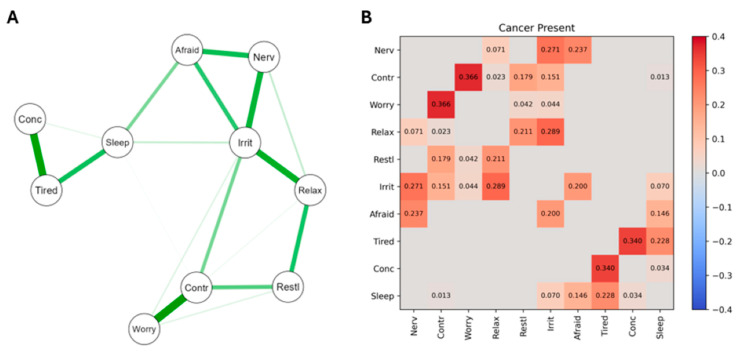
The network structure (**A**) and heatmap (**B**) of significant edge weights correlating GAD symptoms for PCa patients whose cancer was being treated for the first time.

The network structure and heatmap of significant edge weights correlating GAD symptoms for PCa patients whose cancer was present and receiving initial treatment are presented in Figure 1. For the network (Figure 1A), the strongest nodes are at the left of the map, with positive connections indicated by a green line and thicker lines indicating edge weight/connection strength. The correlation values for the significant edge weights are presented numerically on the heatmap in Figure 1B, where red indicates positive correlations and blue negative correlations (there were none of these). Overall, these patients showed the least number of significant connections between GAD symptoms of the three PCa groups studied.

The 10 GAD items were as follows: 1, Feeling nervous, anxious, on edge (Nerv); 2, Unable to control worrying (Contr); 3, Worrying too much about different things (Worry); 4, Trouble relaxing (Relax); 5, Restless (Restl); 6, Easily annoyed, irritable (Irrit); 7, Afraid something awful will happen (Afraid); 8, Easily Tired (Tired); 9, Difficulty concentrating (Conc); 10, Difficulty sleeping (Sleep).

#### 3.2.2. PCa Recurring

**Figure 2:**
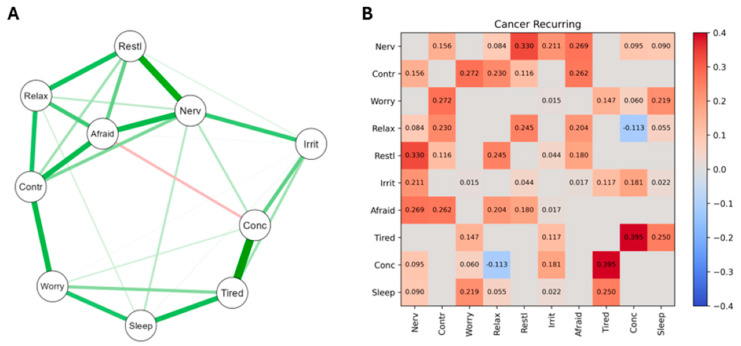
The network structure and heatmap of significant edge weights correlating GAD symptoms for PCa patients whose cancer was recurring.

The network structure and heatmap of significant edge weights correlating GAD symptoms is shown in Figure 2. Actual correlation coefficients are shown in Figure 1B, where red indicates positive correlations and blue negative correlations.

The network structure (A) shows the connections between GAD symptoms for PCa patients whose cancer was recurring. The strongest nodes are at the left of the map, with positive connections indicated by a green line and negative connections indicated by a red line. Thicker lines indicate strong edge weight/connection strength. The heatmap of significant edge weights (B) is shown, with significant positive correlations shown in red and negative correlations shown in blue. Network correlation values are shown within heatmap squares if the edge weight is significant. The 10 GAD items were as follows: 1, Feeling nervous, anxious, on edge (Nerv); 2, Unable to control worrying (Contr); 3, Worrying too much about different things (Worry); 4, Trouble relaxing (Relax); 5, Restless (Restl); 6, Easily annoyed, irritable (Irrit); 7, Afraid something awful will happen (Afraid); 8, Easily Tired (Tired); 9, Difficulty concentrating (Conc); 10, Difficulty sleeping (Sleep).

#### 3.2.3. PCa in Remission

**Figure 3:**
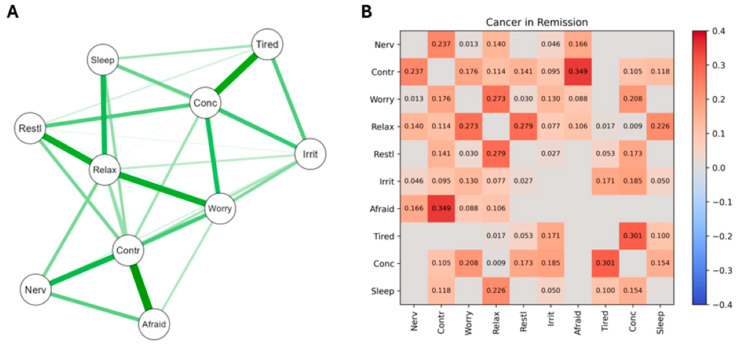
The network structure and heatmap of significant edge weights correlating GAD symptoms for PCa patients whose cancer was in remission.

For PCa patients whose cancer was in remission, the network structure and heatmap of significant edge weights correlating GAD symptoms are presented in Figure 3. Part B presents the actual correlations (red = positive, blue = negative).

The network map (A) indicates the strongest nodes at the left of the map, with positive connections indicated by a green line and negative connections indicated by a red line (there were none). Thicker lines indicate strong edge weight/connection strength. The heatmap of significant edge weights (B) shows the significant positive correlations in red and the significant negative correlations in blue. The 10 GAD items were as follows: 1, Feeling nervous, anxious, on edge (Nerv); 2, Unable to control worrying (Contr); 3, Worrying too much about different things (Worry); 4, Trouble relaxing (Relax); 5, Restless (Restl); 6, Easily annoyed, irritable (Irrit); 7, Afraid something awful will happen (Afraid); 8, Easily Tired (Tired); 9, Difficulty concentrating (Conc); 10, Difficulty sleeping (Sleep).

### 3.3. Comparing Networks

#### 3.3.1. Structural Comparison

Typically, structures of comparative networks can be simply calculated by comparing the numbers of nodes and edges in each network and comparing them [9]. Expanding on those data, the ‘density’ of each network can be found from the ratio of the number of actual edges to the number of possible edges [10]. Because the number of nodes is the same in each of the three PCa patients’ networks (i.e., the 10 GAD-10 items) and the number of total edges is also static, Table 1 presents the density of each network. The GAD symptom network of those PCa patients whose cancer was in remission was the densest, followed by those patients whose PCa was recurring, and then PCa patients who were receiving their first treatment. By reference to the sample sizes, it is apparent that these density ratios were not solely an outcome of how many PCa patients were in each group.

#### 3.3.2. Visualization

The A Sections of Figure 1, Figure 2 and Figure 3 provide valuable information regarding the overall differences in how the GAD-10 symptoms were found to relate to each other across the PCa subgroups. As well as the numerical information already described regarding the comparative density of these networks (Section 3.3.1), viewing Figure 1, Figure 2 and Figure 3 also highlights different subgroup networks of GAD-10 items.

For example, in Figure 1, the strongest node in the network connections was between difficulty concentrating, feeling easily tired, and difficulty sleeping. These patients also displayed strong positive connections between worrying too much, being unable to control worry, and feeling restless. Finally, being easily irritated was a weaker node, but it had the most network connections, showing significant correlations with trouble relaxing, feeling nervous, feeling afraid, and difficulty controlling worries.

In Figure 2, the strongest node was difficulty controlling worries, with strong positive connections with feeling worried, feeling nervous, and feeling afraid, as well as feeling restless and difficulty relaxing. The strongest connection in the network was between feeling tired and difficulty concentrating, and in this group of connections feeling tired also significantly correlated with trouble sleeping and feeling worried. The only negative connection determined in this study was between difficulty relaxing and difficulty concentrating.

In Figure 3, the strongest node was feeling restless, which positively connected with difficulty relaxing, poor concentration, feeling tired, feeling irritable, and difficulty controlling worries. The strongest network connection was between difficulty controlling worries and feeling afraid. Difficulty relaxing was the most connected node in PCa patients with cancer in remission, with significant positive connections to all the GAD symptoms.

#### 3.3.3. Network Comparison Test

The Network Comparison Test [8] uses a complex statistical procedure to identify differences in network structure and specific edge strength across groups. These may be evaluated separately or combined (to give an overall value of comparability). However, as noted by Borkulo et al. [8], differences in sample sizes between groups can reduce the statistical power of these two overall comparison tests to identify genuine inter-group effects at the individual node level. Consequently, the statistical test for differences between the three groups of PCa patients may have had insufficient power to detect actual differences between the networks of the three PCa groups in this case. However, like MANOVA, when sometimes the main effect is nonsignificant but the univariate effects warrant consideration to detect meaningful differences between groups that might otherwise be missed [11], the omnibus network comparison test statistics found here might mask valuable inter-group differences in these three PCa samples, simply due to the power restrictions imposed by differing sample sizes.

With this caveat in mind, the NCT was applied to the three PCa group pairs set out in Table 2. Three statistics are provided for each PCa pair comparison: the Network Invariance Test (signified by the letter M, which compares the overall structure of two networks), the Global Strength Invariance Test (the letter S, which examines the overall strength of the networks (i.e., the total weight of the edges) being compared), and the Edge Invariance Test for each pair of nodes across the two groups being compared (only shown in Table 1 for those pairs of nodes which had a *p* value of <0.07, so as to identify possible trends in edge differences). Because of the differences in sample size across the three PCa groups, the Edge Invariance Test provides the most valuable method of comparing the specific GAD symptom-to-symptom associations that differed between the three PCa groups.

Several of the results reported in column 4 of Table 2 reached acceptable levels of significance, and others were suggestive of trends towards significance, which may have emerged if the sample sizes had been larger and equal. By recourse to Figure 1, Figure 2 and Figure 3, Edge Invariance *p* values shown in Table 2 point to the possible presence of meaningful differences in aspects of GAD across these three groups of PCa patients. These are most easily seen via the heatmaps for Figure 1, Figure 2 and Figure 3.

For example, comparing the heatmap data from the second rows of Figure 1 and Figure 3, PCa patients whose cancer had just been diagnosed and was receiving initial treatment had a nonsignificant edge between the GAD symptoms of worrying too much and having trouble relaxing, but the patients whose PCa had been treated and was now in remission had a significant edge between these two GAD symptoms. Similarly (third row of Table 1), patients whose PCa was receiving its first treatment did not have a significant association between their severity of worrying and their difficulties sleeping, but patients whose PCa was recurring after initial treatment did have a significant edge between these two nodes. An example from the lowest section of Table 1 indicates that patients whose PCa was in remission did not have a significant edge between feeling nervous and feeling restless, but patients whose PCa was recurring did have a significant edge between these two nodes. Other examples of the differences in associations between GAD symptoms (nodes) across the three stages of PCa severity can be observed from the heatmaps in Figure 1, Figure 2 and Figure 3, and Table 2, column 4, and will be discussed below when considering clinical implications arising from these findings.

## 4. Discussion

Apart from the nonsignificant, but potentially invalid, Network Invariance and Global Strength Invariance results from the Network Comparison Test, which may have been due to the large differences in sample sizes, the remaining findings from this study challenge the null hypothesis that was formulated in the Introduction. That is, by examining the three patient groups’ network structures, their visual appearance, and the specific node pairings across the three groups of PCa patients, it may be concluded that the ways that the symptoms of GAD inter-relate with each other differ according to the cancer stage of PCa patients.

### 4.1. Differences Between PCa Groups

There were clear differences in the densities of the three networks, and those differences were not aligned with the sizes of the three PCa groups, suggesting that the complexity of GAD symptom–symptom associations found here was not purely a statistical artifact. While there is no obvious reason from these data why PCa patients whose cancer is in remission should have more complex GAD networks than PCa patients whose cancer is recurring after treatment or patients whose PCa is undergoing initial treatment, there is ample evidence that many PCa patients do suffer from anxiety of cancer recurrence after treatment and that this anxiety is associated with poorer quality of life, plus more symptoms of mental illness [7,12,13]. Although beyond the reach of this study, it may be that PCa patients’ GAD could be fuelled by their social interactions with family and friends who (for example) are worried that, despite the PCa being in remission, it may not be completely cured. Patients who are undergoing their initial treatment for PCa may possess a simpler network of GAD symptoms because they are relatively new to the PCa experience and expect their PCa will be cured.

The differences in GAD symptom–symptom complexity shown in Table 1 were confirmed by the visualization of the three GAD networks (Figure 1, Figure 2 and Figure 3), which identified the strongest nodes in each network. These were fatigue, sleeping difficulties, and poor concentration (for patients undergoing initial treatment); difficulty controlling worries, feeling nervous, and being unable to relax (for those whose cancer was recurring); and restlessness, inability to relax, poor concentration, fatigue, and irritability (when their PCa was in remission). The fourth column in Table 2 extends these findings by identifying those particular pairs of GAD symptoms that were most strongly different in their associations across each pair of PCa subgroups.

### 4.2. Clinical Implications of These Differences

The first (and most important) implication to arise from these findings is that GAD in PCa patients is not able to be validly forced into a ‘one-size-fits-all’ model of treatment, based upon the variability in GAD symptom connections reported above. The need to consider the heterogeneity of GAD when diagnosing anxiety and prescribing treatments is acknowledged [14,15], although the majority of research on heterogeneity in major psychiatric disorders has been conducted with depression [16], with very little on PCa patients’ GAD. Nevertheless, GAD is heterogeneous [17], and PCa patients require differentiation of their treatment based upon their symptom profiles [18].

Secondly, based upon the specific structures, appearances, and Edge Invariance results reported above, it is clear that PCa patients at different stages of their diagnosis–treatment journey need to be considered as requiring differential treatment considerations. Although a discussion of all the possible models of GAD symptom connection differences between the three groups of patients reported in Table 2 would require a more focused and detailed consideration than is possible here, several of those differences do warrant attention as examples of how clinical care might proceed differently.

For example, from Table 2, clinicians might choose to focus upon the connection between the symptoms of worrying too much and an inability to relax if they were treating PC patients whose cancer was in remission, but they would be justified in focusing on worry alone if the PCa patients were receiving their initial treatment. Similarly, patients in remission would be more likely to benefit from treatment focused on their inability to control their worry and its association with feeling afraid, whereas patients whose PCa was receiving its initial treatment might be more likely to respond to treatment focused upon their ability to control their worrying rather than their feelings of fear. These kinds of juxtapositions across pairs of GAD symptoms, with treatment focusing on one rather than both symptoms, could be informed by the data reported in column 4 of Table 2.

### 4.3. Limitations

The major limitation of this study is the disparity in sizes of the three PCa groups. That disparity is probably a reflection of the population of these men, or at least those men who agree to participate in research such as this. Because of its voluntary nature, and due to the fact that most PCa patients do recover (at least initially), it is understandable that more men who were in remission accepted the invitation to take part in this study. Similarly, those whose PCa was defying treatment (i.e., recurring) might be less likely to feel interested in research. The study was cross-sectional, and therefore the relative proportions of the three patient groups reflect the general outcomes of PCa treatment in the geographical areas where patients were recruited. Forced compliance with the study was not applied (for ethical reasons), and the selection of subgroups of patients from (say) the remission group to form more evenly matched groups reduces the representativeness of the sampling process. As a future extension of this study, an investigation of the social interactions and cognitive information processing experienced by these men could assist in understanding how those factors might contribute to the differences in GAD networks between PCa stages. The possible relevance of past psychiatric disorder history could be a valuable inclusion in future studies. In addition, because there is a variety of different treatments that patients can receive (prostatectomy, radiation therapy, hormonal therapy), an examination of the effects of different types of treatment would be a potentially fruitful avenue for further research.

There are some strengths to this study that warrant mention. The GAD-10 was based upon the GAD-7, which has strong validity and reliability but is hindered in its ability to effectively measure all of the diagnostic criteria for GAD. The use of the GAD-10 overcame that limitation, and so the GAD data collected here may be seen as representing all aspects of GAD. Using self-report was also a strength, because interviews conducted by the participants’ treating oncologist, or another mental health professional perceived to be associated with the patients’ oncologists, may have led to positively biased responses. Although most previous research into the profiles of GAD in PCa patients has reported on the bases of a number of symptom–symptom correlations, those procedures do not take into account the interplay between GAD symptoms that is possible from network analysis. Thus, these results provide a unique perspective on how PCa stage is associated with the wide range of GAD symptom–symptom associations.

As a final comment, these data are part of a larger study, parts of which were previously reported concerned with the association between psychological resilience and depression [6]. That is a different research question to the one being investigated here, and the minor overlap between anxiety and depression does not completely justify joining both measures in a single report. However, it should be acknowledged that careful consideration of overall PCa patient mental health needs to include both anxiety and depression as discrete but relevant variables, although such a global discussion was not the focus of this investigation.

## 5. Conclusions

The unique contribution made by this study is for the understanding of how connections between GAD symptoms vary according to the patient’s PCa status, which has not been previously reported. As well as providing some further understanding of the structure of GAD in PCa patients, these results emphasize the need for individualization of clinical assessment and treatment of this often-comorbid psychological disorder in PCa patients.

## Figures and Tables

**Table 1:** Structural comparison for three groups of PCa patients, 10 nodes each.

Network	Density (Actual Edges–Possible Edges)
PCa diagnosed and first treatment (*n =* 71)	17:40 = 0.425
PCa recurring (*n =* 44)	27:40 = 0.675
PCa in remission (*n =* 300)	32:40 = 0.8

**Table 2:** Network Comparison Test results for three pairs of PCa patients.

Pair	Network Invariance	Global Strength Invariance	Edge Invariance (Node Pairs)
PCa being treated vs. PCa in remission	M = 0.349 *p* = 0.404	S = 1.443 *p* = 0.090	Worry–Relax: *p* = 0.050 Nerv–Irrit: *p* = 0.058 Contr–Afraid: *p* = 0.065 Irrit–Afraid: *p* = 0.069
PCa being treated vs. PCa recurring	M = 0.329 *p* = 0.959	S = 1.468 *p* = 0.544	Nerv–Contr: *p* = 0.068 Nerv–Restl: *p* = 0.008 Worry–Sleep: *p* = 0.046
PCa recurring vs. PCa in remission	M = 0.329 *p* = 0.614	S = 0.025 *p* = 0.942	Worry–Relax: *p* = 0.050 Nerv–Restl: *p* = 0.009 Worry–Sleep: *p* = 0.059

GAD-10 abbreviations: Nerv = Feeling nervous, anxious, on edge; Contr = Unable to control worrying; Worry = Worrying too much about different things; Relax = Trouble relaxing; Restl = Restless; Irrit = Easily annoyed, irritable; Afraid = Afraid something awful will happen; Sleep = Difficulty sleeping.

## Data Availability

Dataset available on request from the authors.

## References

[1] Brunckhorst O., Hashemi S., Martic A., George G., Van Hemelrijck M., Dasgupta P., Stewart R., Ahmed K. (2021). Depression, anxiety, and suicidality in patients with prostate cancer: A systematic review and meta-analysis of observational studies. Prostate Cancer Prostatic Dis..

[2] Pan S., Wang L., Zheng L., Luo J., Mao J., Qiao W., Zhu B., Wang W. (2023). Effects of stigma, anxiety and depression, and uncertainty in illness on quality of life in patients with prostate cancer: A cross-sectional analysis. BMC Psychol..

[3] Epskamp S., Borsboom D., Fried E. (2018). Estimating psychological networks and their accuracy: A tutorial paper. Behav. Res. Methods.

[4] Borsboom D., Deserno M.K., Rhemtulla M., Epskamp S., Fried E.I., McNally R.J., Robinaugh D.J., Perugini M., Dalege J., Costantini G. (2021). Network analysis of multivariate data in psychological science. Nat. Rev. Methods Primers.

[5] APA (2022). Diagnostic and Statistical Manual of Mental Disorders.

[6] Sharpley C.F., Arnold W.M., Christie D.R., Bitsika V. (2024). Network connectivity between psychological resilience and depression in prostate cancer patients. Psycho-Oncology.

[7] James C., Brunckhorst O., Eymech O., Stewart R., Dasgupta P., Ahmed K. (2022). Fear of cancer recurrence and PSA anxiety in patients with prostate cancer: A systematic review. Support. Care Cancer.

[8] van Borkulo C., van Bork R., Boschloo L., Kossakowski J., Tio P., Schoevers R., Borsboom D., Waldorp L.J. (2022). Comparing network structures on three aspects: A permutation test. Psychol. Methods.

[9] Coronges K.A., Stacy A.W., Valente T.W. (2007). Structural comparison of cognitive associative networks in two populations 1. J. Appl. Soc. Psychol..

[10] Scott J. (1992). Network Analysis: A Handbook.

[11] Tabachnik B., Fidell L. (2013). Using Multivariate Statistics.

[12] Meissner V.H., Olze L., Schiele S., Ankerst D.P., Jahnen M., Gschwend J.E., Herkommer K., Dinkel A. (2021). Fear of cancer recurrence and disease progression in long-term prostate cancer survivors after radical prostatectomy: A longitudinal study. Cancer.

[13] van de Wal M., van Oort I., Schouten J., Thewes B., Gielissen M., Prins J. (2016). Fear of cancer recurrence in prostate cancer survivors. Acta Oncol..

[14] Nunes A., Trappenberg T., Alda M. (2020). We need an operational framework for heterogeneity in psychiatric research. J. Psychiatry Neurosci. JPN.

[15] Nunes A., Trappenberg T., Alda M. (2020). The definition and measurement of heterogeneity. Transl. Psychiatry.

[16] Park S.-C., Kim Y.-K. (2021). Challenges and strategies for current classifications of depressive disorders: Proposal for future diagnostic standards. Major Depressive Disorder: Rethinking and Understanding Recent Discoveries.

[17] Beard C., Björgvinsson T. (2014). Beyond generalized anxiety disorder: Psychometric properties of the GAD-7 in a heterogeneous psychiatric sample. J. Anxiety Disord..

[18] Gale C., Glue P., Guaiana G., Coverdale J., McMurdo M., Wilkinson S. (2019). Influence of covariates on heterogeneity in Hamilton Anxiety Scale ratings in placebo-controlled trials of benzodiazepines in generalized anxiety disorder: Systematic review and meta-analysis. J. Psychopharmacol..

